# Value of point-of-care ultrasound in the early identification of left ventricular dysfunction and prognostic assessment in cancer patients complicated by sepsis

**DOI:** 10.3389/fonc.2026.1831631

**Published:** 2026-06-05

**Authors:** Junwei Ji, Jiaqi Lian, Guoming Chen, Mei Han, Yaoxian Wang, Liman Yan, Wei Chen, Chuntao Song, Xiaotian Han, Lei Zhao, Bin Yu

**Affiliations:** 1 Department of Emergency Medicine, East Branch, The Fourth Hospital of Hebei Medical University, Shijiazhuang, China; 2 Department of Emergency Medicine, Handan Central Hospital, Handa, China; 3 Department of Ultrasound, The Fourth Hospital of Hebei Medical University, Shijiazhuang, China; 4 Department of Emergency Medicine, The Fourth Hospital of Hebei Medical University, Shijiazhuang, China

**Keywords:** bedside ultrasound, early identification, left heart dysfunction, prognosis, sepsis, tumors

## Abstract

**Background:**

Sepsis-induced myocardial dysfunction (SIMD) significantly contributes to the elevated mortality observed in cancer patients. While point-of-care ultrasound (POCUS) has emerged as a crucial tool for hemodynamic evaluation, its prognostic utility in this specific vulnerable population remains underexplored. This study aimed to assess left ventricular (LV) function using POCUS and determine its prognostic value in cancer patients complicated by sepsis.

**Methods:**

In this retrospective observational study, we enrolled 75 adult cancer patients complicated by sepsis admitted to the Emergency Intensive Care Unit between July 2023 and January 2025. Within 24 hours of sepsis diagnosis, clinical severity scores (SOFA, APACHE II), cardiac biomarkers, and comprehensive POCUS parameters (including LVEF and early diastolic tissue velocity, e’) were recorded. The primary outcome was 28-day survival. Independent predictors were identified using multivariate Cox proportional hazards regression, and prognostic performance was evaluated via receiver operating characteristic (ROC) curves.

**Results:**

Patients presenting with LV dysfunction (LVEF < 50%) exhibited a significantly higher 28-day mortality rate (*p* < 0.05). Multivariate analysis identified the e’ velocity as an independent predictor of 28-day survival (HR = 0.609, 95% CI: 0.454–0.818; p = 0.001). Furthermore, ROC analysis revealed that the combination of e’ and cardiac troponin I (cTnI) demonstrated strong predictive value (AUC = 0.823). The composite prognostic model—integrating e’, cTnI, SOFA, and APACHE II scores—achieved the highest predictive accuracy (AUC = 0.874), outperforming any individual parameter.

**Conclusions:**

Our preliminary findings suggest a potential association between lower POCUS-derived e′ and worse 28-day survival in cancer patients complicated by sepsis. While early e′ measurement may help identify high-risk patients, the current study does not establish a robust prognostic model to guide treatment decisions. These hypothesis-generating results warrant further validation in larger prospective cohorts.

## Introduction

Malignancies have emerged as a major global public health challenge. While advances in therapeutics have improved patient survival rates, the impairment of the immune system caused by both the underlying malignancies and their treatments renders these patients highly susceptible to concurrent infections. Sepsis, one of the most severe complications in cancer patients, is defined as a life-threatening organ dysfunction caused by a dysregulated host response to infection (1). In 1980, Parrillo et al. utilized radionuclide cineangiography to monitor left ventricular (LV) volume and systolic function in patients with severe sepsis, discovering the presence of LV dilation and abnormal systolic performance. This pioneering work led to the concept of sepsis-induced myocardial dysfunction (SIMD) (2). SIMD occurs in a substantial proportion of septic patients and is closely associated with early mortality (3). Furthermore, the mortality rate among septic patients who develop cardiovascular dysfunction is significantly higher than in those without such cardiac impairment (4).

The timely identification of sepsis-induced cardiac dysfunction, coupled with the early implementation of targeted therapeutic interventions, is of paramount importance for improving the prognosis of septic patients. In recent years, point-of-care ultrasound (POCUS) has rapidly advanced in the field of critical care. As an affordable and widely accessible modality, POCUS offers the distinct advantages of being safe, rapid, non-invasive, and capable of bedside execution, making it an indispensable component of contemporary clinical practice. POCUS can visualize alterations in cardiac morphology, wall motion, blood flow, and overall function, allowing for serial patient assessments to continuously optimize therapeutic strategies (5). Sepsis-induced cardiac impairment primarily manifests as reversible LV dysfunction. Studies have demonstrated that myocardial dysfunction in septic patients is intimately linked to inflammatory responses, oxidative stress, and direct myocardial injury. By evaluating specific LV functional parameters, such as the early diastolic tissue velocity (e’) and the E/e’ ratio, POCUS provides crucial insights into the cardiac functional status of these patients (6). Research indicates that echocardiography reveals diastolic dysfunction in approximately 44% of septic patients, which frequently coexists with systolic impairment (7). However, studies regarding the application of POCUS specifically in cancer patients complicated by sepsis remain relatively scarce. In particular, its value in the early identification of LV dysfunction and prognostic assessment within this highly vulnerable subpopulation has not been fully explored.

Therefore, this study applied POCUS to evaluate LV function in cancer patients complicated by sepsis, aiming to investigate its prognostic value and provide a structured clinical rationale for the early identification of this critical condition.

## Methods

### Study design and setting

This retrospective observational study was conducted in the Emergency Intensive Care Unit (EICU) of the Fourth Hospital of Hebei Medical University. The study protocol was approved by the institutional medical ethics committee (Approval No.: 2025KT019). Given the retrospective nature of the study, the requirement for written informed consent was waived by the ethics committee.

### Study population

We screened adult patients (≥ 18 years of age) diagnosed with both malignancy and sepsis who were admitted to the EICU between July 1, 2023, and January 30, 2025. Sepsis was defined according to the Third International Consensus Definitions for Sepsis and Septic Shock (Sepsis-3) (8). Patients were required to have a hospitalization duration of more than 24 hours with complete medical records. To eliminate confounding factors affecting baseline cardiac function, we strictly excluded patients with pre-existing organic heart diseases (e.g., congenital heart disease, valvular heart disease, or cardiomyopathy), hematological malignancies, and those with a history of anthracycline chemotherapy (doxorubicin, daunorubicin, epirubicin, idarubicin) or direct cardiac radiation. Additionally, pregnant or lactating women, patients receiving end-of-life hospice care or who had treatment withdrawn, those with incomplete ultrasound data, and cases lost to follow-up for the 28-day survival outcome were excluded from the final analysis. Ultimately, 75 eligible patients were enrolled in the study.

### Clinical data collection and grouping

Comprehensive clinical data were extracted, including demographics (age, sex), vital signs at diagnosis (temperature, heart rate, respiratory rate, blood pressure, mean arterial pressure), primary tumor site, infection source, and underlying comorbidities. Laboratory parameters within 24 hours of sepsis diagnosis were recorded, encompassing white blood cell (WBC) count, N-terminal pro-B-type natriuretic peptide (NT-proBNP), cardiac troponin I (cTnI), myoglobin (MYO), procalcitonin (PCT), and blood lactate levels. Disease severity was assessed using the Sequential Organ Failure Assessment (SOFA) and Acute Physiology and Chronic Health Evaluation II (APACHE II) scores.

Patients were stratified into two groups based on left ventricular ejection fraction (LVEF): the normal LV function group (LVEF ≥ 50%) and the LV dysfunction group (LVEF < 50%). For prognostic evaluation, the cohort was further divided into survivor and non-survivor groups based on the 28-day survival outcome.

### Point-of-care ultrasound protocol and quality control

Within 24 hours of a confirmed sepsis diagnosis, all patients underwent bedside echocardiographic evaluation to assess left ventricular function. The POCUS parameters acquired included LVEF, left ventricular outflow tract (LVOT) diameter, LVOT velocity-time integral (VTI), stroke volume (SV), and cardiac output (CO). Diastolic function was evaluated by measuring the early diastolic transmitral flow velocity (E wave) and the early diastolic mitral annular tissue velocity (e’, calculated as the average of the septal and lateral annular velocities), thereby deriving the average E/e’ ratio.

In this study, all POCUS data were retrieved from the electronic medical record (EMR) system of the Emergency Intensive Care Unit (EICU). These bedside examinations were performed as part of standard clinical care by experienced intensivists who receive regular formal training and ongoing supervision from specialized ultrasound physicians. To ensure the reliability of the measurements, all POCUS acquisitions in our department are conducted following the standard protocols recommended by the American Society of Echocardiography (ASE).

### Statistical analysis

Statistical analyses were performed using SPSS version 27.0. Continuous variables with a normal distribution were presented as mean ± standard deviation and compared using the Student’s t-test. Non-normally distributed continuous variables were expressed as median (interquartile range) and compared using the Mann-Whitney U test. Categorical variables were presented as frequencies and percentages, with differences assessed by the Chi-square test. Survival distributions between the normal LV function and LV dysfunction groups were estimated using the Kaplan-Meier method and compared via the log-rank test. To identify independent predictors of 28-day mortality, univariate and subsequent multivariable Cox proportional hazards regression models were constructed. The proportional hazards (PH) assumption for the Cox model was verified using Schoenfeld residuals, and no significant violations were observed. To prevent model overfitting due to the limited sample size, we employed a dimension reduction strategy by utilizing comprehensive clinical scores (SOFA and APACHE II) rather than entering individual demographic or comorbidity variables. Additionally, the variance inflation factor (VIF) was used to evaluate the multicollinearity among the variables eventually included in the multivariable Cox model. Receiver operating characteristic (ROC) curves were generated to evaluate the predictive value of individual and combined indicators for the 28-day survival outcome. A two-sided *p* value < 0.05 was considered statistically significant.

## Results

### Baseline characteristics and clinical data

A total of 75 adult cancer patients complicated by sepsis were enrolled in this study, comprising 37 patients in the normal LV function group (LVEF ≥ 50%) and 38 patients in the LV dysfunction group (LVEF < 50%). The cohort included 46 males (24 in the normal LV group, 22 in the LV dysfunction group) and 29 females (13 in the normal LV group, 16 in the LV dysfunction group), with no significant difference in gender distribution between the two groups (*p* > 0.05). The mean age of patients in the LV dysfunction group was significantly higher than that in the normal LV group (*p* < 0.05). However, there were no significant differences between the two groups regarding primary tumor site, source of infection, chronic comorbidities, or history of anti-tumor therapy within the preceding 30 days (*p* > 0.05).

Regarding vital signs, the heart rate (HR) in the LV dysfunction group was significantly elevated compared to the normal LV group (*p* < 0.05). Laboratory analyses revealed that the median levels of NT-proBNP and cTnI were significantly higher in the LV dysfunction group (*p* < 0.05). Furthermore, the mean APACHE II score was significantly higher in the LV dysfunction group (*p* < 0.05), whereas the SOFA score showed no significant inter-group difference (*p* > 0.05). Most importantly, the 28-day mortality rate was significantly higher in the LV dysfunction group compared to the normal LV function group (*p* < 0.05) (**Table 1**).

**Table 1:** Baseline characteristics and clinical data of patients.

Variables	Total (n = 75)	Normal LV function (n = 37)	LV dysfunction (n = 38)	*P* value
Male, n (%)	46 (61.30)	24 (64.80)	22 (57.80)	0.535
Female, n (%)	29 (38.70)	13 (35.20)	16 (42.20)	
Age (mean ± SD)	65.43 ± 10.91	62.84 ± 10.00	67.95 ± 11.29	0.042
Primary tumor site, n (%)				
Upper gastrointestinal tract	10 (13.40)	4 (10.80)	6 (15.80)	0.768
Lower gastrointestinal tract	13 (17.40)	4 (10.80)	9 (23.60)	0.234
Biliary tract	4 (5.40)	2 (5.40)	2 (5.30)	1
Breast	2 (2.60)	1 (2.70)	1 (2.70)	1
Liver	6 (8.00)	5 (13.50)	1 (2.70)	0.190
Lung	16 (21.30)	8 (21.70)	8 (21.00)	1
Pancreas	7 (9.30)	5 (13.50)	2 (5.30)	0.406
Uterine and adnexal	6 (8.00)	1 (2.70)	5 (13.10)	0.214
Others	11 (14.60)	7 (18.90)	4 (10.05)	0.483
With anti-tumor therapy within 30 days, n (%)	38 (50.70)	19 (51.40)	19 (50.00)	0.907
Without anti-tumor therapy within 30 days, n (%)	37 (49.30)	18 (48.60)	19 (50.00)	
Source of infection, n (%)				
Respiratory tract	26 (34.70)	9 (27.30)	17 (44.70)	0.063
Abdominal cavity	16 (21.30)	7 (18.90)	9 (23.70)	0.615
Biliary tract	10 (13.30)	8 (21.60)	2 (5.30)	0.081
Intestinal tract	3 (4.00)	1 (2.70)	2 (5.20)	1
Bloodstream	26 (34.70)	14 (37.80)	12 (31.60)	0.569
Urinary tract	2 (2.70)	0 (0.00)	2 (5.30)	0.485
Others	8 (10.70)	5 (13.50)	3 (7.90)	0.679
Underlying comorbidities, n (%)				
Hypertension	29 (42.60)	18 (54.50)	11 (31.40)	0.054
Diabetes mellitus	15 (22.10)	7 (21.20)	8 (22.80)	0.870
Coronary heart disease	10 (14.70)	6 (18.10)	4 (11.40)	0.658
Others	10 (14.70)	4 (12.10)	6 (17.10)	0.672
Clinical scores				
SOFA score, median (IQR)	6.00 (4.00,9.00)	5.00 (4.00,8.50)	7.00 (4.00,9.25)	0.306
APACHE II score (mean ± SD)	16.95 ± 6.29	13.97 ± 5.51	17.89 ± 6.46	0.006
Vital signs				
Temperature, median (IQR)	37.60 (37.30, 38.00)	37.60 (37.35, 37.90)	37.60 (37.30, 38.35)	0.643
Heart rate (mean ± SD)	107.95 ± 23.21	102.14 ± 25.42	113.61 ± 19.54	0.031
Respiratory rate, median (IQR)	20.00 (18.00, 24.00)	20.00 (18.00, 22.00)	20.00 (18.75, 25.25)	0.303
MAP (mean ± SD)	84.01 ± 15.85	87.00 ± 13.93	81.11 ± 17.20	0.108
Laboratory findings				
WBC, median (IQR)	8.69 (4.28, 14.07)	10.84 (5.66, 14.67)	7.18 (3.74, 10.03)	0.273
PCT, median (IQR)	21.00 (6.00, 55.00)	20.00 (3.15, 36.50)	31.00 (11.5, 66.25)	0.060
Lactate (mean ± SD)	2.81 ± 1.74	2.45 ± 1.65	3.16 ± 1.78	0.075
NT-proBNP, median (IQR)	389.00 (100.00, 4690.00)	100.00 (100.00, 1425.00)	2910.00 (100.00, 8050.00)	0.013
MYO, median (IQR)	50.00 (50.00, 50.00)	50.00 (24.50, 50.00)	50.00 (50.00, 214.50)	0.106
cTnI, median (IQR)	0.01 (0.01, 0.39)	0.01 (0.01, 0.01)	0.01 (0.01, 0.09)	0.002
28-day mortality, n (%)	29 (38.70)	8 (21.60)	21 (55.30)	0.003

### Echocardiographic parameters

Comparisons of POCUS parameters demonstrated no significant differences in E wave velocity and LVOT diameter between the two groups (*p* > 0.05). Conversely, the mean values of e’, VTI, SV, and CO were significantly lower in the LV dysfunction group than in the normal LV group, while the median E/e’ ratio was significantly higher in the LV dysfunction group (*p* < 0.05) (**Table 2**).

**Table 2:** Comparison of echocardiographic parameters between the two groups.

Variables	Normal LV function	LV dysfunction	*P* value
E wave (mean ± SD)	77.89 ± 20.07	74.57 ± 27.12	0.813
e', median (IQR)	9.83 (8.90,11.69)	6.71 (6.10,7.43)	<0.001
E/e' ratio, median (IQR)	7.61 (5.88,8.90)	10.69 (7.23,13.67)	<0.001
LVOT diameter (mean ± SD)	1.83 ± 0.22	1.75 ± 0.23	0.127
VTI (mean ± SD)	20.77 ± 2.18	19.14 ± 3.25	0.012
SV (mean ± SD)	55.95 ± 14.96	46.92 ± 15.37	0.012
CO (mean ± SD)	5.59 ± 1.66	4.52 ± 1.25	0.003

### Survival analysis

**Figure 1:**
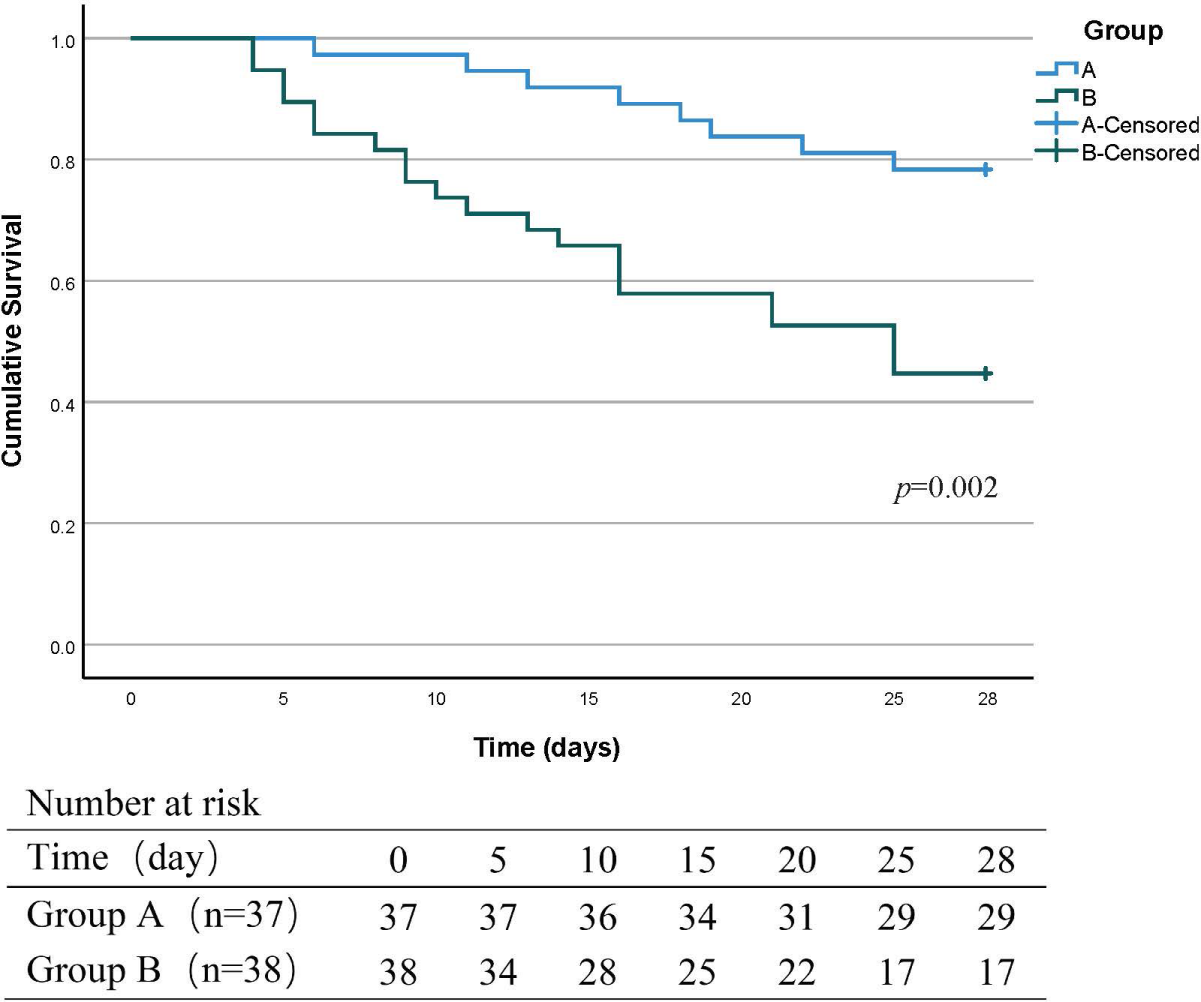
Kaplan-Meier survival curves of patients in different left ventricular function groups. Group A: normal LV function (LVEF ≥ 50%); Group B: LV dysfunction (LVEF < 50%). Crosses (+) indicate censored cases. Survival was significantly lower in Group B compared to Group A (Log-rank test, *p* = 0.002).

Kaplan-Meier survival analysis was performed to compare the 28-day survival outcomes between the two groups. The resulting survival curve demonstrated a statistically significant decrease in survival for patients with LV dysfunction compared to those with normal LV function (95% CI: 16.372–23.154; Log-rank *p* = 0.002) (**Figure 1**).

### Prognostic value assessment and independent predictors

Based on the 28-day outcome, the cohort was divided into a survivor group (n = 46) and a non-survivor group (n = 29). Univariate analysis revealed significant differences between survivors and non-survivors in e’ (*p* < 0.001), E/e’ (*p* = 0.001), VTI (*p* = 0.001), SV (*p* = 0.009), CO (*p* = 0.025), SOFA score (*p* < 0.001), APACHE II score (*p* < 0.001), lactate (*p* < 0.001), cTnI (*p* = 0.005), and respiratory rate (*p* < 0.001). Conversely, univariate Cox regression confirmed that demographics (age, sex) and most underlying comorbidities (e.g., diabetes mellitus, coronary heart disease) were not significantly associated with 28-day survival, with the exception of hypertension (**Tables 3**, **4**).

**Table 3:** Comparison of clinical and echocardiographic parameters between survivors and non-survivors.

Variables	Survivors (n = 46)	Non-survivors (n = 29)	*P* value
Male, n (%)	27 (58.70%)	19 (65.50%)	0.555
Female, n (%)	19 (41.30%)	10 (34.50%)	
Age (mean ± SD)	65.23 ± 9.08	65.27 ± 13.49	0.865
Primary tumor site, n (%)			
Upper gastrointestinal tract	6 (13.00%)	4 (13.8%)	1
Lower gastrointestinal tract	5 (10.90%)	8 (27.60%)	0.064
Biliary tract	3 (6.50%)	1 (3.40%)	0.961
Breast	1 (2.20%)	1 (3.40%)	1
Liver	4 (8.70%)	2 (6.90%)	1
Lung	8 (17.40%)	8 (27.6%)	0.294
Pancreas	4 (8.70%)	3 (10.30%)	1
Uterine and adnexal	6 (13.00%)	0 (0.00%)	0.112
Others	9 (19.60%)	2 (6.90%)	0.240
With anti-tumor therapy within 30 days, n (%)	26 (56.50%)	12 (42.40%)	0.201
Without anti-tumor therapy within 30 days, n (%)	20 (43.50%)	17 (58.60%)	
Source of infection, n (%)			
Respiratory tract	9(19.56%)	10(34.48%)	0.148
Abdominal cavity	10(21.73%)	5(17.24%)	0.859
Biliary tract	7(15.21%)	4(13.79%)	1
Intestinal tract	2(4.30%)	5(17.24%)	0.144
Bloodstream	13(28.26%)	8(27.58%)	0.949
Urinary tract	2(4.30%)	0(0.00%)	0.687
Others	2(4.30%)	2(6.89%)	1
Underlying comorbidities, n (%)			
Hypertension	20(43.47%)	8(27.58%)	0.166
Diabetes mellitus	12(26.08%)	5(17.24%)	0.543
Coronary heart disease	10(21.73%)	4(13.79%)	0.578
Others	3(6.52%)	1(3.44%)	0.961
Clinical scores			
SOFA score, median (IQR)	4.50(3.75,6.00)	8.00(7.00,11.00)	<0.001
APACHE II score (mean ± SD)	13.37 ± 5.16	20.03 ± 5.79	<0.001
Vital signs			
Temperature, median (IQR)	36.60(36.30,37.00)	36.60(36.25,37.35)	0.810
Heart rate (mean ± SD)	104.00 ± 23.79	114.20 ± 21.15	0.063
Respiratory rate, median (IQR)	19.00(17.00,20.00)	25.00(22.00,29.00)	<0.001
MAP (mean ± SD)	84.84 ± 16.20	82.68 ± 15.45	0.569
Laboratory findings			
WBC, median (IQR)	7.76(3.98,12.69)	11.54(4.88,17.44)	0.063
PCT, median (IQR)	18.50(4.60,39.00)	35.00(8.50,64.00)	0.063
Lactate (mean ± SD)	2.19 ± 0.92	3.80 ± 2.23	<0.001
NT-proBNP, median (IQR)	130.50(100.00,3505.00)	1630.00(100.00,6688.50)	0.164
MYO, median (IQR)	50.00(43.75,50.00)	50.00(50.00,335.50)	0.150
cTnI, median (IQR)	0.12(0.12,0.12)	0.13(0.12,0.23)	0.005
Echocardiographic parameters			
E wave (mean ± SD)	73.43 ± 20.29	78.06 ± 28.55	0.414
e', median (IQR)	9.01(7.40,11.32)	6.80(6.10,7.99)	<0.001
E/e' ratio, median (IQR)	7.60(5.85,10.15)	10.56(7.43,13.71)	0.001
LVOT diameter (mean ± SD)	1.82 ± 0.25	1.73 ± 0.10	0.109
VTI (mean ± SD)	20.76 ± 2.64	18.64 ± 2.78	0.001
SV (mean ± SD)	55.09 ± 16.27	45.48 ± 13.06	0.009
CO (mean ± SD)	5.37 ± 1.51	4.55 ± 1.50	0.025
LVEF, median (IQR)	57.00(49.00,60.25)	50.00(48.50,58.00)	0.065

**Table 4:** Univariate and multivariate cox proportional hazards regression analyses for predicting 28-day survival.

Variables	Univariate analysis		Multivariate analysis	
	HR (95% CI)	*P* value	HR (95% CI)	*P* value
Age	1.004 (0.968 - 1.041)	0.846	–	–
Sex	1.196 (0.556 - 2.574)	0.647	–	–
E wave	1.007 (0.992 - 1.023)	0.360	–	–
e'	0.592 (0.457 - 0.766)	<0.001	0.609 (0.454 - 0.818)	0.001
E/e' ratio	1.176 (1.086 - 1.274)	<0.001	–	–
LVOT diameter	0.275 (0.055 - 1.371)	0.115	–	–
VTI	0.767 (0.656 - 0.897)	0.001	–	–
SV	0.967 (0.943 - 0.992)	0.011	–	–
CO	0.705 (0.532 - 0.932)	0.014	–	–
LVEF	0.955 (0.911 - 1.001)	0.057	–	–
SOFA score	1.166 (1.072 - 1.268)	<0.001	1.067 (0.899 - 1.267)	0.456
APACHE II score	1.112 (1.057 - 1.170)	<0.001	1.029 (0.922 - 1.149)	0.610
cTnI	1.722 (1.064 - 2.787)	0.027	1.088 (0.610 - 1.941)	0.774
NT-proBNP	1.000 (1.000 - 1.000)	0.101	–	–
MYO	1.001 (1.000 - 1.002)	0.077	–	–
Hypertension	0.330 (0.146 - 0.747)	0.008	–	–
Diabetes mellitus	0.732 (0.279 - 1.920)	0.526	–	–
Coronary heart disease	0.773 (0.269 - 2.221)	0.632	–	–

Prior to constructing the multivariable Cox regression model, potential multicollinearity among the significant predictors was carefully assessed. To avoid structural collinearity, highly interdependent hemodynamic parameters (e.g., SV, CO, and VTI) were intentionally excluded. The Variance Inflation Factor (VIF) values for the remaining selected variables (e’, cTnI, SOFA, and APACHE II) were all strictly less than 5, indicating no severe multicollinearity. Subsequently, to prevent overfitting while adjusting for baseline disease severity, a multivariable Cox proportional hazards model was constructed incorporating these four variables. Crucially, this analysis confirmed that e’ (HR = 0.609, 95% CI: 0.454–0.818; *p* = 0.001) remained an independent predictor of 28-day survival in cancer patients complicated by sepsis.

### Predictive performance of individual and combined indicators

Receiver operating characteristic (ROC) curves were constructed to evaluate the predictive value of various parameters for the 28-day survival outcome. For individual indicators, e’ demonstrated an optimal cutoff value of 7.45 cm/s, yielding an AUC of 0.811 (sensitivity 86.2%, specificity 78.3%). The APACHE II score had an optimal cutoff of 14.50 points (AUC 0.808, sensitivity 89.7%, specificity 65.2%), and the SOFA score had an optimal cutoff of 6.50 points (AUC 0.775, sensitivity 79.3%, specificity 78.3%). cTnI alone was not a strong predictor (AUC 0.666).

**Figure 2:**
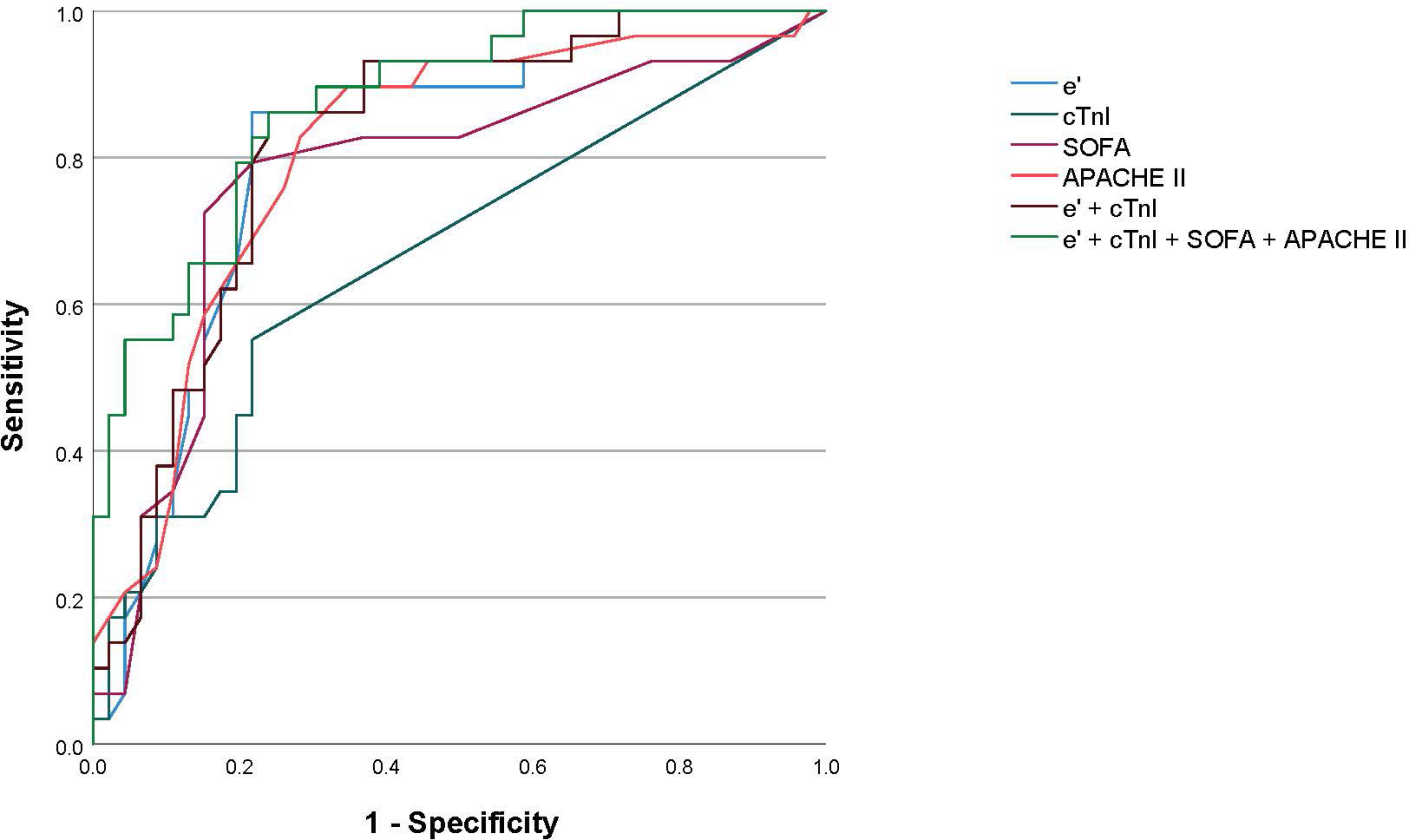
Receiver operating characteristic (ROC) curves of individual parameters and their combinations for predicting 28-day survival. The composite model (e’+cTnI+SOFA+APACHE II) demonstrated the highest predictive value (AUC = 0.874). cTnI, cardiac troponin I; SOFA, Sequential Organ Failure Assessment; APACHE II, Acute Physiology and Chronic Health Evaluation II.

Notably, the predictive performance improved when combining indicators. The combination of e’ and cTnI yielded an AUC of 0.823 (sensitivity 86.2%, specificity 76.1%). The most robust predictive model was the composite of e’, cTnI, SOFA score, and APACHE II score, which achieved the highest AUC of 0.874, with a sensitivity of 86.2% and a specificity of 76.1% (**Table 5**, **Figure 2**).

**Table 5:** Predictive value of individual parameters and their combinations for 28-day survival.

Variables	AUC	*P* value	95% CI	Optimal cutoff	Sensitivity (%)	Specificity (%)	Youden index
e'	0.811	<0.001	0.711-0.912	7.45	86.2	78.3	0.645
cTnI	0.666	0.016	0.536-0.795	0.01	55.2	78.3	0.334
SOFA score	0.775	<0.001	0.658-0.891	6.50	79.3	78.3	0.576
APACHE II score	0.808	<0.001	0.706-0.911	14.50	89.7	65.2	0.549
e' + cTnI	0.823	<0.001	0.727-0.919	–	86.2	76.1	0.623
e' + cTnI + SOFA + APACHE II	0.874	<0.001	0.796-0.952	–	86.2	76.1	0.623

## Discussion

Despite increasingly profound research and advances in the management of sepsis, its overall incidence continues to rise, and it remains the leading cause of mortality in Intensive Care Units (ICUs) and emergency departments. Epidemiological data indicate that approximately one in five hospitalized sepsis patients has an underlying malignancy (9). In the first year following a cancer diagnosis, the incidence of sepsis is approximately 3.7%, with roughly one-third of these patients progressing to septic shock, carrying a staggering mortality rate of 35.5% (10). This heightened vulnerability is largely attributed to intensive anti-tumor therapies (e.g., surgery, stem cell/bone marrow transplantation, chemotherapy, and radiotherapy) (11), with varying tumor sites and treatment modalities differentially impacting sepsis mortality (12).

Sepsis is characterized by a dysregulated host immune response to infection, leading to life-threatening organ dysfunction. The massive release of pro-inflammatory cytokines induces vasodilation and increased capillary permeability, resulting in the extravasation of intravascular fluid. When these cytokines act upon the myocardium, they provoke a severe yet reversible cellular “shock,” defined clinically as sepsis-induced myocardial dysfunction (SIMD) (13). Reports suggest that SIMD occurs in nearly 60% of septic patients (14). Therefore, the timely identification of sepsis-induced cardiac dysfunction and the early implementation of targeted interventions are of paramount importance for improving prognosis. As the frontline of critical care, the emergency department must prioritize the early recognition and proactive management of this highly lethal complication.

Echocardiography, an imaging modality utilizing ultrasound waves to assess cardiac structure and function, offers distinct advantages over traditional invasive hemodynamic monitoring techniques, such as pulmonary artery catheterization (PAC) or Pulse Contour Cardiac Output (PiCCO) monitoring. It is intuitive, accurate, non-invasive, and highly reproducible. Point-of-care ultrasound (POCUS) has long been advocated as a comprehensive and rapid non-invasive approach for the cardiac assessment of hemodynamically unstable patients (15). Using the MIMIC database, Feng et al. (16) demonstrated that the application of transthoracic echocardiography during an ICU stay was associated with a reduced risk of sepsis-related mortality. Conversely, a retrospective cohort study by Blank et al. (17) found no association between echocardiography within the first five days of admission and a reduction in 28-day mortality, though the fundamental diagnostic and therapeutic utility of ultrasound was not negated. Despite a scarcity of prospective studies, echocardiography remains extensively utilized in ICUs worldwide (18).

LVEF is the most commonly utilized parameter for evaluating left ventricular (LV) systolic function in clinical practice. However, its association with mortality in sepsis and septic shock remains controversial. A retrospective study by Dugar et al. (19) revealed a U-shaped relationship, where both severe LV systolic dysfunction (LVEF < 25%) and hyperdynamic LVEF (≥ 70%) were independently associated with higher in-hospital mortality. Similarly, a multicenter prospective cohort study by Delgado-Serrano et al. (20) demonstrated that patients with severe diastolic dysfunction (and low filling pressures) had the poorest prognosis, whereas LVEF itself was not significantly associated with elevated sepsis mortality. Consistent with these findings, our study utilized an LVEF of 50% as a cutoff; while the 28-day mortality was significantly higher in the LV dysfunction group, LVEF was not an independent predictor of 28-day survival overall.

Stroke volume (SV) and cardiac output (CO) are critical parameters in sepsis management to ensure adequate tissue oxygen delivery. While massive fluid resuscitation is often required to augment CO and tissue perfusion, it carries the risk of fluid overload, which can precipitate pulmonary edema, right ventricular failure, elevated central venous pressure, and subsequent organ congestion (21). In our study, significant differences in both SV and CO were observed between the survivor and non-survivor groups, highlighting the value of early echocardiographic functional assessment in guiding fluid therapy and avoiding deleterious fluid overload.

Compared to the E wave and the E/A ratio, which are heavily influenced by loading conditions, the early diastolic mitral annular tissue velocity (e’) measured by Tissue Doppler Imaging (TDI) is less load-dependent (22). An E/e’ ratio > 8 is widely considered a robust indicator of diastolic dysfunction (23). A meta-analysis by Sanfilippo et al. (24) confirmed a strong correlation between low e’ velocities, high E/e’ ratios, and increased mortality in severe sepsis. Our findings corroborate this evidence; the non-survivor group exhibited significantly lower e’ and higher E/e’ values. Furthermore, multivariate analysis established e’ as an independent predictor of 28-day survival in cancer patients complicated by sepsis.

Cardiac biomarkers, including cTnI and NT-proBNP, provide complementary diagnostic value. While NT-proBNP was significantly elevated in our LV dysfunction group, it did not significantly differ between survivors and non-survivors. This may be attributed to confounding factors affecting NT-proBNP synthesis and clearance, such as renal dysfunction, massive fluid resuscitation, and mechanical ventilation (25). Conversely, cTnI levels were significantly different between survival groups. Notably, the combination of e’ and cTnI demonstrated excellent predictive value, and the composite model (e’ + cTnI + SOFA + APACHE II) achieved the highest AUC, providing a promising prognostic tool for this vulnerable patient population.

This model offers a potential framework for clinicians to identify high-risk patients during the early stages of sepsis by integrating cardiac function indicators (low e′), evidence of myocardial injury (elevated cTnI levels), and overall disease severity (high SOFA/APACHE II scores). For these high-risk patients, physicians may implement the following targeted strategies in addition to standard treatment: First, adopt a restrictive fluid management approach, utilizing precise volume assessments guided by left ventricular diastolic function to prevent cardiac deterioration caused by fluid overload. Second, initiate or optimize vasoactive drug therapy at an early stage to reduce cardiac load while ensuring adequate organ perfusion. Third, intensify monitoring and care, including expedited admission to the ICU, more frequent dynamic POCUS evaluations, and proactive management of complications. Through these interventions, the model is anticipated to translate predictive capabilities into tangible improvements in clinical outcomes, facilitating a crucial paradigm shift from merely “predicting mortality” to actively “guiding patient survival.”

This study has several limitations. First, as a single-center retrospective study with a relatively small sample size (75 patients and only 29 outcome events), the results may be subject to selection bias. Consequently, the events-per-variable (EPV) ratio is limited. Furthermore, the composite model was developed and evaluated within the same cohort without internal or external validation; thus, the risk of model overfitting cannot be completely excluded, and the reported predictive performance (such as the AUC) must be interpreted cautiously, warranting future large-scale prospective validations. Second, the inherent heterogeneity of malignancies—including variations in tumor site, staging, and prior treatments—may influence outcomes; specifically, due to sample size constraints, we could not perform a granular subgroup analysis on the distinct cardiotoxic effects of specific immunochemotherapy regimens. Third, patients with pre-existing heart failure with preserved ejection fraction (HFpEF) were not explicitly excluded. Consequently, elevated NT-proBNP in patients with LVEF >50% may partially reflect acute sepsis-induced stress superimposed on chronic diastolic dysfunction. Fourth, while the POCUS data were reliably obtained by experienced critical care physicians, our study only analyzed baseline parameters, and due to the retrospective design, formal interobserver or intraobserver variability analyses for POCUS measurements were not available. Since sepsis-induced myocardial dysfunction is often reversible within 7 to 10 days (26), future research should be designed to repeatedly collect POCUS data and to examine the trajectories of echocardiographic indices at 3 days, 5 days, 7 days, and even at one-month follow-up in patients. Fifth, although no severe multicollinearity was detected among the variables in our final model (all VIFs < 5), concerns regarding potential collinearity and the limited sample size restricted the simultaneous inclusion of all echocardiographic parameters (such as VTI, SV, and CO) in the multifactor Cox model. Future studies with larger sample sizes are needed to further verify the independent predictive effects of these specific hemodynamic markers. Finally, all measurements were obtained via transthoracic echocardiography (TTE), which, compared to transesophageal echocardiography (TEE), is subject to image quality limitations in emergency settings (e.g., due to patient obesity). Therefore, while we advocate prioritizing feasible markers like e′, technically demanding parameters (e.g., VTI, SV) should be considered optional depending on the patient’s acoustic window.

## Conclusion

Our preliminary findings suggest a potential association between lower POCUS-derived e′ and worse 28-day survival in cancer patients complicated by sepsis. While early e′ measurement may help identify high-risk patients, the current study does not establish a robust prognostic model to demonstrate improved clinical outcomes or guide definitive treatment decisions. These hypothesis-generating results provide a foundation for early risk stratification, but warrant further validation in larger, prospective, multicenter cohorts.

## Data Availability

The raw data supporting the conclusions of this article will be made available by the authors, without undue reservation.
